# Glial-Neuronal Interactions in Pathogenesis and Treatment of Spinal Cord Injury

**DOI:** 10.3390/ijms222413577

**Published:** 2021-12-17

**Authors:** Nadezda Lukacova, Alexandra Kisucka, Katarina Kiss Bimbova, Maria Bacova, Maria Ileninova, Tomas Kuruc, Jan Galik

**Affiliations:** Institute of Neurobiology, Biomedical Research Centre, Slovak Academy of Sciences, Soltesovej 4–6, 040 01 Kosice, Slovakia; kisucka@saske.sk (A.K.); bimbova@saske.sk (K.K.B.); bacova@saske.sk (M.B.); ileninova@saske.sk (M.I.); kuruc@saske.sk (T.K.); galik@saske.sk (J.G.)

**Keywords:** microglia and astrocytes phenotypes, intercellular crosstalk, lesion microenvironment, neuroinflammation, in vivo glia-to neuron reprogramming, subpial delivery, gut dysbiosis, electrostimulation, rehabilitation, neuroprotective strategies

## Abstract

Traumatic spinal cord injury (SCI) elicits an acute inflammatory response which comprises numerous cell populations. It is driven by the immediate response of macrophages and microglia, which triggers activation of genes responsible for the dysregulated microenvironment within the lesion site and in the spinal cord parenchyma immediately adjacent to the lesion. Recently published data indicate that microglia induces astrocyte activation and determines the fate of astrocytes. Conversely, astrocytes have the potency to trigger microglial activation and control their cellular functions. Here we review current information about the release of diverse signaling molecules (pro-inflammatory vs. anti-inflammatory) in individual cell phenotypes (microglia, astrocytes, blood inflammatory cells) in acute and subacute SCI stages, and how they contribute to delayed neuronal death in the surrounding spinal cord tissue which is spared and functional but reactive. In addition, temporal correlation in progressive degeneration of neurons and astrocytes and their functional interactions after SCI are discussed. Finally, the review highlights the time-dependent transformation of reactive microglia and astrocytes into their neuroprotective phenotypes (M2a, M2c and A2) which are crucial for spontaneous post-SCI locomotor recovery. We also provide suggestions on how to modulate the inflammation and discuss key therapeutic approaches leading to better functional outcome after SCI.

## 1. Introduction

Spinal cord injury (SCI) is one of the most devastating events leading to serious neurological deficits. The complex pathophysiology of SCI, consisting of primary and secondary mechanisms, may explain the difficulty in finding a suitable therapy [1]. Traumatic SCI is caused by several distinct events, which follow a somewhat overlapping temporal sequence: the acute phase (seconds to minutes after injury), the secondary phase (minutes to weeks after injury), and the chronic phase (months to years after the injury) [2]. After direct mechanical insult, the spinal tissue undergoes a cascade of cellular and molecular events which exacerbate the primary lesion. Secondary injury includes disruption of the vasculature and increased blood-spinal cord permeability, ischemia, local edema, ionic imbalance, inflammation, cell death (necrosis and apoptosis), and activation of inhibitory molecules followed by demyelination and axonal degeneration [3,4]. While neurons and glia die at the lesion site (approximately 2–4 mm) within minutes and hours after SCI, cell types, including neurons, astrocytes, microglia and oligodendrocytes surrounding the lesion site are lost in a delayed manner [5,6].

Inflammatory response is one of the key mechanisms of secondary injury. It includes activation of resident cells (microglia, astrocytes) and recruitment of immune cells (macrophages and neutrophils) from the bloodstream to the injury site. Resident and immune cells release proinflammatory cytokines, including interleukins (IL-1, IL-6) and tumor necrosis factor-α (TNFα), all of which increase the extent of the inflammatory response. These events play an important role in secondary tissue damage and cell death. After SCI, many factors act simultaneously on macrophages and microglia. Naïve astrocytes become reactive; they produce inhibition molecules, such as proteoglycans and laminin in the extracellular space. Reactive astrocytes then invade the region surrounding the lesion center and lead to glial scar formation. While the glial scar limits regeneration and acts pathologically as a physical and biochemical barrier to axonal regeneration [7,8], the transformation of reactive astrocytes into their polarization states in the subacute phase serves some neuroprotective function [9]. Previous findings suggest that in the early stages of SCI reactive astrocytes can play a crucial role in neuroprotection through limiting the apoptosis of neuronal cells, providing trophic support to neurons and maintaining homeostasis [10,11]. Anderson et al. [12] demonstrated that the presence of glial scar is required for axonal regeneration and that the prevention of glial scar formation impairs the regenerative processes. They supported the hypothesis that reactive forms of astrocytes show remarkable diversity in their expression gene expression, signaling mechanisms and morphology.

To develop appropriately targeted repair strategies, there is a need for detailed understanding of how various cell populations interact with each other within the lesion site and in the surrounding spinal cord tissue in both acute and subacute phases of SCI. This review focuses on microglia-astrocyte crosstalk and glial-neuronal interactions in an acute inflammatory response which involves numerous cellular and non-cellular components. We pay special attention to activation of microglia and astrocytes phenotypes in the acute and subacute phase after SCI and highlight the neuroprotective effects of microglial and astroglial polarization on the functional outcome. Some other promising strategies for spinal cord repair will be discussed separately.

## 2. Neutrophils and Monocytes Respond Early after SCI

The response to spinal cord trauma is mediated by multiple coordinated molecular pathways, which are activated soon after SCI within the lesion site and spread throughout the spinal cord in spatio-temporal manner. After SCI, neutrophils infiltrate the epicenter of injury and produce neurotoxic effects by promoting the expression of inducible nitric oxide synthase *(*iNOS) and cyclo-oxygenase 2 **(**COX-2), and releasing pro-inflammatory cytokines [13,14,15]. Since neurons and glia synthesize pro-inflammatory cytokines, e.g., tumor necrosis factor (TNF)-α and interleukin 1β (IL-1β), as part of normal intercellular communication [16], their release after SCI evokes inflammation and causes dysregulation of cytokine release leading to the death of neurons and oligodendrocytes [17]. Recently, a ~12-fold increase in IL-1β level has been reported 4 h after Th9 compression in rats [18]. The level of this cytokine rapidly decreased one day after SCI, however, apoptosis appeared in cell populations all around the lesion site (5 mm from the lesion epicenter). The authors detected activation of caspase-3 in neurons, astrocytes and oligodendrocytes, but not in microglial cells.

Cytokines are potentially noxious molecules and together with chemokines are mainly produced by microglia, astrocytes, and peripheral macrophages. In addition to TNFα and IL-1β, other molecules such as interleukin 1*α* and 6 (IL-1*α,* IL-6*)*, leukemia inhibitory factor (LIF), nitric oxide (NO), reactive oxygen species (ROS), elastase and matrix metalloproteinase-9 (MMP-9) are released [19]. After spinal trauma, two waves of cellular infiltration occur- first wave consists of polymorphonuclear leukocytes (PMN) activated mainly by IL-1, IL-2 and IL-6. The second wave is characterized by the presence of macrophages and monocytes recruited via various chemokines such as chemokine (C-C motif) ligand 2 (CCL2), chemokine (C-X-C motif) ligand 1 (CXCL1) and chemokine (C-X-C motif) ligand 2 (CXCL2). The balance between the proinflammatory vs. anti-inflammatory effects of these signaling proteins plays a crucial role in the progression of SCI [20].

Blood-derived monocytes are also massively recruited at the lesion site, and they play a dual role (i.e., they remove cell debris and repair injured spinal cord tissue [21] as well as producing neurotoxic factors [22]). After SCI, monocytes differentiate into macrophages and adopt many of the markers and behaviors of microglia. Since the similarities between monocyte-derived macrophages (MDMs) and microglia have complicated the development of efficient prediction tools to discriminate between them, they are sometimes still referred to as microglia/macrophages [23]. A very recent study by Kisucká et al. [9] demonstrated that microglial/macrophage marker Cd11b was markedly expressed at the lesion site (3 mm) and in adjacent spinal cord tissue (3 mm cranially and caudally) one week after Th9 compression.

## 3. Microglia Rapidly Accumulate around the Lesion Site and Influences Neurons and Astrocytes in the Subacute Phase after SCI

Traumatic SCI results in a dysregulated microenvironment which is largely driven by the immediate and robust response of resident astrocytes and microglia [24] leading to neuronal death. Min et al. [6] studied the discrete roles of microenvironment- regulating cells in rat SCI model (Th9 contusion) at the lesion site (approximately 2 mm), where neurons acutely died immediately post-SCI, and in two penumbra regions (P1 and P2). In P1 area, immediately surrounding the lesion, the neurons underwent death between 12 h and 1d, while in P2 area neurons remained healthy for up to two weeks. The authors found that ramified ionized calcium-binding adaptor molecule 1 (Iba-1+) cells (resident microglia) died earlier than neurons in P1 area. Interestingly, neurons remained healthy in region where microglia was morphologically activated. Round Iba-1+ cells with strong expression of CD45 (identified as glia and/or infiltrated blood cells) appeared after neurons had died, and expressed phagocytic activity. The evident connection between microglia activation and delayed neural death was not confirmed. The authors suggest that Iba-1+ cells, including ramified and round cells, are innocent in delayed neuronal death. They speculate that loss of the supportive function of astrocytes may contribute to delayed neuronal death. Bellver-Landente et al. [23] studied the response of microglia in a mouse model of SCI (Th10-Th11 contusion) and found extensive microglial proliferation during the first week post-SCI. The authors discovered that microglia formed a dense cellular interface at the border of the lesion between reactive astrocytes and infiltrating MDMs, referred to as the “microglial scar”. Depletion of microglia after SCI using PLX5622 (CSF1R inhibitor which crosses the blood–spinal cord barrier) reduced the number of neurons and oligodendrocytes at the injury site, disrupted the organization of the astrocytic scar and impaired functional outcome. Accordingly, the central nervous system (CNS) delivery of microglial proliferation factor M-CSF at the site of contusion boosted microglial proliferation and enhanced locomotor recovery.

These data indicate that proliferating microglia are a key cellular component of the microglial scar which develops during the first week post-SCI to protect neural tissue. In the light of our recently published data, the first week post-injury is critical for modulation of reactive microglia/astrocytes into their neuroprotective phenotypes [9].

## 4. Protection of Microglial Phenotypes after SCI

Microglia respond to SCI by activating and then evolving into M1 (pro-inflammatory) or M2 (anti-inflammatory) phenotypes [9,25]. There is growing evidence that mechanisms responsible for the neuroprotective functions of activated microglia include several functional behaviors [26]. One of the most important ways in which microglia could contribute to neuroprotection is synaptic stripping, a process in which microglia selectively remove inhibitory synapses from injured neuronal perikarya [27]. This intimate interaction between microglia and synapses is associated with motoneuron regeneration [28], promotion of neuronal survival [29] and reduction of neuronal cell death [30]. It is also well known that microglia actively promote neurogenesis following CNS injury through producing insulin-like growth factor-1, which suppresses apoptosis and increases proliferation and differentiation of neural stem cells [31]. Activated microglia may also boost neurogenesis by means of an unconventional mechanism through provoking non-committed oligodendrocyte progenitor cells to adopt a neuronal phenotype [32]. Another essential mechanism of neuroprotective microglial function is microglial phagocytosis, a process necessary for maintaining CNS homeostasis [33,34,35]. Moreover, microglia can suppress neuroinflammation, restore homeostasis, and protect nerve tissues by producing anti-inflammatory cytokines and cytoactive factors for repairing tissue [36].

Currently, there is direct evidence, that the neuroprotective environment after SCI is associated with the alternatively-activated, proliferating phenotypes of M2 microglia. These microglial phenotypes play an important role in the healing process by sustaining homeostasis and dampening inflammation, resulting in the release of neurotrophic factors and anti-inflammatory cytokines to promote tissue sparing and functional recovery after SCI, and this effect persists for five weeks after SCI [23]. Microglial M2 phenotypes can be categorized into M2a, M2b, M2c and M2d subtypes. M2a, b and c phenotypes are considered as anti-inflammatory repair microglial cells, and they can be distinguished by observing the changes in expression of the relevant markers. The M2a subtype is responsible for tissue repair and regeneration by expressing anti-inflammatory and immuno-regulatory molecules. This phenotype is activated by interleukin-4 (IL-4) and interleukin-13 (IL-13), which inhibit the production of pro-inflammatory molecules after SCI, resulting in the upregulation of arginase-1 (Arg-1) and CD206 [37,38].

M2b and M2c microglia are largely phagocytic. M2b microglia involves T-cells recruitment and is activated by Toll-like receptors (TLRs), playing a key role in the innate immune system and immune complexes, resulting in the expression of high levels of anti-inflammatory cytokines (IL-1 and IL-10) and low levels of IL-12. M2c subtype is also involved in inflammation dampening and healing and is activated by a potent anti-inflammatory cytokine IL-10 and glucocorticoids, resulting in high transforming growth factor beta (TGF-β) expression [39,40]. M2d microglia, unlike the above mentioned subsets of alternatively-activated microglia are induced by “switching” from a classically-activated inflammatory phenotype to an alternatively-activated anti-inflammatory/pro-angiogenic phenotype. The M2d subtype originates from the M1 pro-inflammatory phenotype through the activation of adenosine A2A receptors [41].

**Figure 1:**
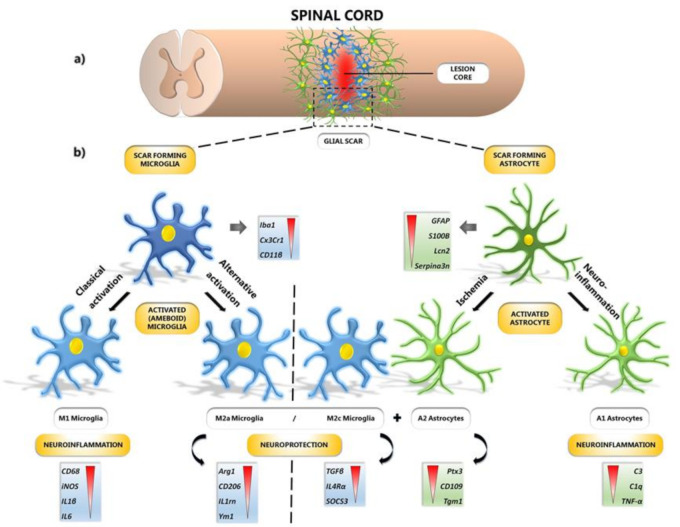
Formation of glial scar after SCI. (**a**) Spinal cord with illustrated lesion core surrounded by microglia and astrocytes, forming a glial scar. (**b**) Focusing on specific types of glial cells: microglia and astrocytes with their corresponding genes ranked according to expression level. Resting microglia and astrocytes acquire scar-forming phenotypes through their activation under certain conditions; they differentiate into several subtypes involved either in neuroinflammation (M1, A1) or neuroprotection (M2a, M2c, A2). The M1 phenotype of microglia is acquired by classical activation, whereas M2a and M2c phenotypes are acquired by alternative activation pathways. Astrocytes, which are differentiated into A2 phenotype under ischemic conditions, promote neuronal survival and tissue repair. A1 phenotype is acquired via secretion of neuroinflammatory markers. *Iba1*—ionized calcium-binding adaptor molecule 1; *Cx3Cr1*—fractalkine receptor; *Cd11β*—beta-integrin marker of microglia; *GFAP*—glial fibrillary acidic protein; *S100B*—calcium-binding protein B; *Lcn2*—lipocalin 2; *Serpina3n*—serine (or cysteine) peptidase inhibitor; *CD68*—cluster of differentiation 68; *iNOS*—inducible nitric oxide synthase; *IL-1β*—interleukin-1β; *IL-6*—interleukin-6; *Arg-1*—arginase-1; *CD206*—mannose receptor and C-type lectin; *IL1rn*—interleukin 1 receptor antagonist; *Ym1*—chitinase-like protein-1; *TGF-β*—transforming growth factor beta; *IL4Rα*—interleukin 4 receptor alpha; *SOCS3*—suppressor of cytokine signaling 3; *Ptx3*—pentraxin 3; *CD109*—cluster of differentiation 109; *Tgm1*—transglutaminase 1; *C3*—complement 3; *C1q*—complement component 1q; *TNF-α*—tumor necrosis factor-alpha; SCI—spinal cord injury.

Spinal cord lesions produce an inhibitory microenvironment which is not in favor of the M2 phenotypes, so the M1 phenotype dominates [42]. Recent evidence suggests that gene expression of anti-inflammatory M2a microglia (*CD206*, *Ym1*—chitinase-like protein-1, *Il1rn*—interleukin 1 receptor antagonist, *Arg-1*), M2c microglia (*TGF-β*, *SOCS3*—suppressor of cytokine signaling 3, *IL4R α*—interleukin 4 receptor alpha) and A2 astrocytes (*Tgm1*—Transglutaminase 1, *Ptx3*—Pentraxin 3, *CD109*—cluster of differentiation 109) is significantly overactivated at the lesion site one week after SCI [9]. The authors also found positive correlation between neurological outcome and the expression of neuroprotective microglia and astrocytes phenotypes. These results provided evidence for the first time that modulation of reactive microglia/astrocytes into their neuroprotective phenotypes contributes to spontaneous locomotor recovery after SCI. Molecular changes leading to functional remodeling could be identified by the use of a set of microglia and astrocyte-specific markers (Figure 1).

## 5. Early Modulation of Inflammatory Response after SCI

The timing of the modulation of inflammatory response after SCI has been of great interest to many researchers over the last few years. Despite advancements in understanding of the pathophysiological mechanisms of secondary inflammation in the spinal cord, treatment options have remained limited in this area. The rationale for modulation of the inflammatory response includes the potential for decreasing the massive spread of the injury which occurs after this traumatic event.

Several recent studies have pointed out the importance of early post-SCI alleviation of the inflammatory response. Zhao et al. [43] found that *XIST* (a cancer-related gene which participates in the development of SCI) was upregulated after spinal trauma in rats (in vivo) and lipopolysacharide (LPS)-activated microglia (in vitro). Authors studied potential interaction between *XIST*, miR-27a and *Smurf1*. Knockdown of *XIST* with lentivirus vectors containing sh-XIST immediately after SCI suppressed cell apoptosis (via decreased expression of *Bcl*-2, *Bax* and cleaved *caspase 3*) and inflammatory response (via reduced expression of *TNF-α* and *IL-6*) probably through sponging of miR-27a and downregulating *Smurf1* in vivo and in vitro. They found that *Smurf1* was the functional gene underlying *XIST*/miR-27a axis during spinal trauma. *Smurf1* could partially reverse the inhibitory effect of *XIST* knockdown on the cell death and inflammation. These results implicate a novel *XIST*/miR-27a/*Smurf1* pathway included in SCI. Papa et al. [44] demonstrated that inhibitory treatment of microglia with minocycline-loaded nanoparticles (NPs), applied immediately after SCI, induced a major long-lasting effect up to 63 days post injury, confirming the relevant pro-inflammatory effect of activated microglial cells in the earliest stages of degeneration after spinal trauma. They noticed that early after SCI (from one to seven days) the amount of microglial cells (CD11b^pos^/CD45^low^) rapidly increased. Recruited macrophages (CD11b^pos^/CD45^high^) were barely detected at the first day post-SCI and peaked at the seventh day. Interestingly, authors studied the uptake of minocycline-loaded nanoparticles and they found out, that at the first day post SCI was the uptake of NPs high in microglial cells while very low in macrophages. At the seventh day after spinal trauma NPs uptake were still very low in infiltrated macrophages. The quantitative evaluation of the NPs uptake ratio between microglia and recruited macrophages showed that predominantly microglial cells were affected by this treatment. It is well known that IL-1β is a main pro-inflammatory cytokine in the spinal cord, producing a harmful microenvironment in injured tissue and amplifying the extent of the injury. An antagonist to IL-1β receptor has also been shown to alleviate the actions of IL-1β by decreasing the severity of neuronal damage, reducing cell death and improving motor function [45]. Our study also pointed out the importance of early modulation of the inflammatory response after traumatic SCI. A single dose of 3-hydroxyl-3-methylglutaryl-coenzyme A reductase (HMG-CoA) inhibitor Atorvastatin (ATR, 5 mg/kg, i.p.) applied immediately after spinal trauma significantly reduced IL-1β levels (almost to control level), decreased microglial activation in the dorsolateral area, inhibited macrophage infiltration into the white and gray matter and significantly decreased the expression of apoptotic markers 24 h after Th9 compression [18]. The therapeutic benefit of ATR has been presented in several other studies addressing SCI. These studies monitored long-term administration vs. single dose of ATR via per-oral and intraperitoneal application [46,47,48,49,50,51,52]. However, the most effective method for ATR administration proved to be early, intraperitoneal injection.

As mentioned above, macrophages/microglia may initiate pathological secondary mechanisms, and on the other hand they can promote regeneration of traumatized spinal cord based on their phenotype (destructive M1 vs. beneficial M2 status) [53]. One day after SCI we observed significant increase in gene expression of both phenotypes; however the expression of M1 prevailed over the M2 phenotype. ATR significantly reduced both M1 and M2 phenotypes at the epicenter of injury and in the adjacent cranial segment. Since ATR modulated the M1 phenotype more markedly than the M2 antigenic marker, we assume that the neuroprotective effect of ATR could lie in their polarization. In addition, marked activity of a pro-apoptotic protein- caspase-3 was noticed throughout the whole injured area in neurons and glial cells (astrocytes and oligodendrocytes). Atorvastatin treatment visibly reduced the cleavage of caspase-3 and acted as a neuroprotective agent in neuronal and glial cells [18]. Sohn et al. [54] showed that another HMG-CoA- inhibitor called simvastatin effectively decreased cytotoxicity and spinal cord neuronal death due to ischemia–reperfusion injury, probably via moderation of oxidative stress. In this study, simvastatin was applied from the beginning of oxygen and glucose deprivation in vitro and was maintained during the following 24-h reoxygenation period. The authors demonstrated that simvastatin (0.1 to 10 µM) significantly lowers the values for lactate dehydrogenase (LDH), a determinant of cytotoxicity, compared to injured controls (*p* < 0.001). Moreover, the DCFDA (2′,7′-dichlorofluorescein diacetate) value measuring cell redox state (or oxidative stress) was effectively reduced by the simvastatin treatment. Liang et al. [55] used simvastatin (10 mg/kg) in combination with ezetimibe (cholesterol-reducing drug) in three doses during the first 72 h after weight- drop spinal cord injury. They pointed out that the combination of these agents could improve the neurological score and attenuate the endothelial inflammatory response after SCI in rats.

Essentially, the acute inflammatory response following spinal trauma is a crucial element for amplification, spreading and chronicity of the injury [56]. As mentioned above, immediate modulation of the inflammatory response could be an important step towards more successful treatment of traumatic SCI.

## 6. Changes in Gut Microbiota Resulting from SCI as a Trigger of Inflammatory Response

Novel data show that SCI sets in a motion a systematic breakdown of communication between the nervous system, immune system and gastrointestinal system [57]. When the spinal cord is injured, axons which normally descend from brain/brainstem regions to control spinal sympathetic neurons are lost or damaged. The subsequent loss of normal sympathetic tone throughout the body leads to chronic immune dysfunction and gut dysbiosis which can contribute to the development of intraspinal and systemic pathology [57,58,59,60,61,62,63].

Changes in gut permeability induced by trauma can liberate commensal bacteria from the gut lumen, allowing microbes and their metabolites to enter the circulation and trigger inflammation throughout the body [61]. Various genes encoding transcription factors or epithelial tight-junction proteins which regulate paracellular permeability or the proliferation and differentiation of epithelia are dramatically affected by SCI (e.g., *Tcf712*—Transcription factor-7-like, *Cdx1*—Caudal Type Homeobox 1, *Cdx2*—Caudal Type Homeobox 2, *Jam2*—Junctional Adhesion Molecule 2, etc.). In the neurogenic bowel, impaired intestinal transit limits the delivery of important nutrients to the microbiota in the distal colon, and altered mucin production impairs production of the mucus layer which is colonized by gut microorganisms creating a biofilm [57]. Changes in relative abundance of certain gut bacteria induced by SCI correlate with locomotor and immune functions as well [61]. In mice with SCI-induced dysbiosis, exacerbated lesion pathology and intraspinal inflammation (enhanced CD11b+ CNS macrophage response at the lesion epicenter and total number of infiltrating CD3+ T cells and CD45R+ B cells) has been observed. Similarly, changes in GALT (gut-associated lymphoid tissue) immune cell composition (e.g., B220+ cells, CD8+ T cells, CD11c+ cells, CD11b+ macrophages found in mesenteric lymph nodes by 3dpi) occurred in parallel with increased expression levels of pro- and anti-inflammatory cytokines (TNF-α, IL-1β, TGF-β, IL-10) seven days post-injury. O’Connor et al. [62] found significantly elevated pro-inflammatory cytokines (IL-12, MIP-2—macrophage inflammatory protein 2, TNF-α) in the rat intestine four weeks post-SCI. They also found a correlation between cytokine levels (IL-1β, IL-12, MIP-2) and differences in gut microbiota diversity at eight week post-SCI.

In conclusion, the composition of gut microflora significantly affects many physiological processes in the body and the overall health of the host. Currently, there are several studies focusing on analyses of the direct impact of SCI-induced gut dysbiosis on immune and neural functions in rodent models, and also on finding possible therapeutic approaches for regulating inflammation induced by SCI via remodeling of the gut microbiome. Nevertheless, the precise molecular mechanisms participating in the gut/CNS/immune system axis including receptors, their down-stream molecules or transcription factors are still not fully understood.

## 7. Promising Modulation Strategies of SCI-Induced Microglial Plasticity and Gut Dysbiosis

One of the therapeutic tools for managing SCI-induced inflammatory events in the gut could be the application of health-promoting probiotic bacteria with the potential to modulate the gut microflora and thus contribute to restoration of intestinal immune homeostasis. In generally, recognition of probiotic bacteria via TLRs in intestinal dendritic cells leads to their maturation and to release of cytokines, which coordinate the differentiation of naive T-helper cells (Th0) into mature Th1, Th2 or Th3/Treg subpopulations [64,65]. Probiotic bacteria do not cause inflammation, because they can regulate the immune response via a complex of mechanisms including reduction of some TLRs, inhibition of nuclear factor kappa B (NF-κB) and mitogen-activated protein kinase (MAPK) signaling pathways, and induction of TLR-negative regulators [66,67,68]. Application of probiotic Lactobacilli to SCI mice triggered a protective immune response in GALT and improved locomotor recovery [61]. Since the number of immunoregulatory Treg lymphocytes (CD4+CD25+FoxP3+ T cells) and CD11c+ dendritic cells in mesenteric lymph nodes was increased and the lesion volume and axon/myelin pathology at the injury epicenter was reduced, it is reasonable to assume that the gut-CNS-immune axis could play a crucial role in regulation of functional post-SCI recovery.

Another therapeutic approach in this area could be the application of ω-3 polyunsaturated fatty acids (ω-3 PUFAs), that are known to potentiate the immunomodulatory properties of probiotic lactobacilli via stimulation of bacterial adhesion to the intestinal wall [69,70] through their direct effect on the binding sites on the epithelial cells [71] and they are able to modulate TLR/NF-κB/cytokine level and proportion of lymphocyte subsets in the gut [67,68]. In the context of neurotrauma, supplementation of ω-3 PUFAs reduced oxidative stress, apoptosis and the levels of inflammatory mediators (TNFα, IL6) in rats with SCI [72]. Marinelli et al. [73] have observed that ω-3 PUFAs are able to restore behavioral responses and induce pro-regenerative effects and neuroprotective action against demyelination, apoptosis and neuroinflammation in innovative mouse model mimicking human-like features of SCI. In general, the prophylactic properties of ω-3 PUFAs (pre-SCI) include the stabilization of neuron cell membranes, the reduction of the expression of inflammatory cytokines, the improvement of local blood flow, reduced eicosanoid production, activation of protective intracellular transcription pathways, and increased concentration of lipids, glycogen, and oligosaccharides by neurons. On the other hand, the therapeutic properties (post-SCI) include the increased production of endogenous antioxidants such as carnosine and homocarnosine, the maintenance of elevated glutathione concentrations at the site of injury, reduced concentrations of oxidative stress marker malondialdehyde (MDA), autophagy improvement, and p38 MAPK (mitogen-activated protein kinase) expression reduction in the superficial dorsal horns (limiting the sensation of neuropathic pain) [74]. Moreover, Lu et al. [75] have found that ketogenic diet attenuates oxidative stress and inflammation after spinal cord injury by activating nuclear factor-E2 related factor 2 (Nrf2) and suppressing the NF-κB signaling pathways. Spinal cord injury-induced gut dysbiosis could be alleviated also due to melatonin treatment [76] and by treatment with a fecal transplant as well [77]. Furthermore, electroacupuncture modulates the intestinal microecology to improve intestinal motility in spinal cord injury rats [78]. Moreover, Schmidt et al. [79] have showed that that minocycline augments inflammation and anxiety-like behavior following SCI in rats through action on the gut microbiota. In conclusion, there are many approaches to mitigating the impact of spinal cord injury using microflora modulation. However, the precise mechanism is still unclear.

## 8. Interactions in Pathogenesis, Maintenance and Escalation of SCI-Induced Neuropathic Pain, and Development of Therapy

SCI-induced neuropathic pain (NP) remains a significant and disabling clinical problem with very few therapeutic treatment options available. Chronic spinal cord injured patients show several deviations from normal nociception and pain sensitivity, ranging from hyperalgesia (exaggerated sensitivity to noxious stimuli), allodynia (pain in response to normally innocuous stimuli), dysesthesias (disturbing somatic sensation that may not be painful) and spontaneously occurring pain [80,81]. Although some of the therapeutic strategies provide pain relief at—and/or below—injury level hypersensitivity, the effects of exogenous pharmacologic agents are usually temporary. In addition, long-term treatments for chronic pain may be limited in sustained effectiveness, dose escalation, development of tolerance and systemic side effects of medication during prolonged use [82]. Currently, pain treatment includes pharmacologic and psychological options, but these are most suited to provide short-term relief [83,84]. There is a need for improved therapeutic approaches for long-term management of chronic pain.

Most therapeutic approaches focus on a single target that often serves a single function, predominantly neurotransmission. Glutamate excitotoxicity via signaling through *N*-methyl-D-aspartate glutamate (NMDA) receptors is one of the key events in the onset and maintenance of the neuropathic pain. Previous experimental studies have shown decreased hyperexcitability and cutaneous hypersensitivity with intrathecal administration of NMDA receptor antagonists [85,86]. Despite of their modulatory quality, the clinical use of systemic NMDA antagonists is dose-limited by adverse effects such as hallucinations and motor dysfunction [87,88]. Early studies have shown that the naturally occurring peptide histogranin [89] and its stable analog Serine-Histogranin (SHG) have NMDA receptor antagonist activity [90,91,92,93,94]. Similarly, Yu et al. [95] reported that [-]-huperzine A (HUP-A), a naturally occurring Lycopodium alkaloid isolated from the Chinese club moss, *Huperzia serrata*, with potent reversible inhibitory action on NMDA receptors and Acetylcholine Esterase (AchE), offers an exceptional prospect for multimodal treatment of SCI-induced neuropathic pain in rats. NMDA antagonists have also been shown to prevent development of morphine tolerance and allow prolonged administration of other potent analgesics, opioids [96,97]. Opioids have been used in treatment of chronic pain, however their chronic use is accompanied by tolerance and risk of addiction [98,99]. Although cholinergic agonists for pain control are poorly tolerated in general, because of their severe side effects on the gastrointestinal system [100], several pharmacological studies demonstrated that coadministration of AchE inhibitors can potentiate opioid analgesia [81].

Combined administration of NMDA antagonist and opioid receptor agonist [82,101,102] and/or cholinergic agonists [103,104] and antagonists of NMDA-subtype glutamate receptors [103] may be beneficial especially for the long-term pain management. HUP-A treatment (intraperitoneally or intrathecally) after moderate static compression at Th10 level restored homeostasis of central sensory neurocircuitry without invoking drug tolerance and dependence or respiratory suppression [95]. This naturally occurring Lycopodium alkaloid had a cholinergic inhibitory action on dorsal horn pain transmission, possibly through the activation of muscarinic M2 and M4 receptors (mAchRs) on GABAergic inhibitory neurons that mitigate the release of primate afferent-derived glutamate in the dorsal horn [105,106]. The decrease in nociceptive neurotransmission likely was mediated also by the observed immunoreactivity decrease in substance P from primary afferents onto second-order dorsal horn neurons. Cholinergic signaling through mAChRs has been reported to decrease the release of substance P in the rat dorsal spinal cord in response to acute noxious stimulation of the tail [107] and to inhibit pain-transmitting spinothalamic and thalamic neurons [108,109]. Rats treated with intrathecal HUP-A also demonstrated significant reduction in activation of the immune response in microglia and other immune cells, and reduced release of inflammatory cytokines, reactive astrocytes and reactive oxygen species. CD86 (expressed by M1 macrophages), glial fibrillary acidic protein GFAP (marker of astrocytes) and iNOS (a critical neuroinflammatory mediator expressed by immune cells and microglia) [110] were reduced significantly in cervical and lumbar regions of the HUP-A-treated group compared with controls. CD68, a marker for activated microglia and macrophages was reduced in lumbar spinal cord. The oligodendrocyte markers (CNPase) and myelin basic protein (MBP) demonstrated significantly increased preservation of myelin in the lumbar white matter of HUP-A-treated rats, suggesting that intrathecal HUP-A treatment did not trigger myelin toxicity, as has been reported for some prototype NMDA antagonists [84]. Arginase1, expressed by M2 macrophages that have a beneficial role after SCI, was comparable in the cervical and lumbar spinal cord in both HUP-A-treated and saline-treated groups. Moreover, the authors did not find a significant difference in immunoreactivity levels of neurofilament M or H at lesion site from HUP-A-treated and control rats. All these findings support the hypothesis that chronic administration of HUP-A does not introduce further toxicity in the injured spinal cord, and that HUP-A treatment, although not triggering axonal regeneration, protects myelin against the chronic damage resulting from neuroinflammation that is mediated largely by locally activated microglia and astroglia and by macrophage invasion [95].

## 9. Astrocytes Response to SCI

Under physiological conditions, astrocytes mediate CNS homeostasis and provide trophic and metabolic support for neurons regulating synaptic signaling or plasticity [111,112], and they control the cerebrovascular tone and modulate local blood flow [113,114]. It is well established that astrocytes in the white and gray matter are morphologically distinct. Astrocytes in the gray matter are regionally specialized, reflecting for instance the specific neurotransmitter system of that area [115]. White matter astrocytes have distinct functional properties that are necessary for secure saltatory conduction. Previously, Kozlova and Lukanidin [116] reported that astrocytes in spinal cord express the calcium-binding protein Mts1/S100A4. This protein has been shown to influence cell motility, proliferation, angiogenesis, and neurite outgrowth and neuron survival [117,118]. Since S100A4 was up-regulated after peripheral nerve or dorsal root injury exclusively in affected white matter [116], it may be an important regulator of de- and remyelination. Recently, Escartin et al. [119] defined reactive astrocyte nomenclature and pointed out that astrocyte phenotypes should be specified by a combination of molecular markers (not only by GFAP alone) and by functional readouts, predominantly in in vivo conditions. Accumulating evidence suggests that GFAP is predominantly expressed in white-matter astrocytes while S100β tends to be expressed in the astrocytes located in the gray matter [120,121].

In response to SCI, astrocytes undergo multiple morphological and functional changes in the process of reactive gliosis [122]. They migrate towards the lesion site within the first hours after injury [123,124] and proliferate for 24 h with a peak after 48 h [125]. This initial response is necessary to reestablish the blood-brain barrier and restrict further migration or proliferation that prevent the lesion site to expand into surrounding healthy tissue [126,127,128,129]. Subsequently, astrocytes rapidly increase the expression of intermediate filaments of GFAP, vimentin [128,130] and other astrocyte-specific markers [9,119], and they release molecules limiting spontaneous axon sprouting and inhibit regeneration [7,131,132,133,134]. In the acute phase of SCI, naïve astrocytes became reactive and after further proliferation they transformed into a glial scar forming astrocytes [135]. The use of a lentivirus-mediated herpes simplex thymidine kinase/ganciclovir (HSV1tk/GGV) system, in which suicide gene expression was regulated by hGFAP promotor to selectively ablate reactive proliferating astrocytes in a mouse crush injury model, showed impeded glial scar formation, exacerbated neuroinflammation, increased loss of neurons and failure of spontaneous functional recovery [136]. Astrocytic scar in the chronic stages of SCI, as the final form of reactive astrogliosis is widely regarded as a principal cause of axonal re-growth failure and poor functional outcome [137].

## 10. Microglial and Astrocyte Polarization after SCI

For a long time it was not clear whether reactive astrocytes were harmful or beneficial [138]. Liddelow and Barres, [39] and Liddelow et al. [139] previously reported that activation of microglia by classical inflammatory mediators can convert astrocytes into a neurotoxic A1 phenotype in a variety of neurological diseases. These findings pointed to the key role of molecules secreted by activated microglia in the induction of reactive astrocytosis. One week post-SCI, the gene expression of microglia/macrophages and M1 microglia (pro-inflammatory phenotype) was strongly upregulated at the lesion site (3 mm area) and caudally (3 mm), but attenuated afterwards [9]. The common astrocytes (GFAP and S100B) and reactive astrocytes (A1 phenotype) were profoundly expressed predominantly at the lesion site and cranially (3 mm area) two weeks post-SCI. However, gene expression of anti-inflammatory M2a and M2c microglia, as well as A2 astrocytes, which are responsible for up-regulation of neurotrophic factors was greatly activated at the lesion site one week post-SCI [9].

The application of suitable anti-inflammatory drugs inhibiting the formation of A1 astrocytes induced by activated neuroinflammatory microglia could be used as a potential therapeutic agent for the injured spinal cord. Similarly, increased presence of M2 microglial phenotypes at the lesion site might represent a promising strategy for tissue regeneration after SCI [140]. At present, the microglia–astrocyte conversation which ensures their tight reciprocal modulation after SCI is an undeniable fact, and as our recently-published experimental data show, time-dependent regulation of M1/M2 polarization (the expression of M2a, M2c markers) and A1/A2 polarization at the lesion site and 3 mm cranially and/or caudally from the injury epicenter is key for functional outcome after SCI [9].

## 11. In Vivo Conversion of Astrocytes to Neurons

The spatial and temporal patterns of astrocyte and neuron death are similar one week post-SCI [6]. Since it is known that the spinal cord lacks the ability to produce new neurons in adulthood, neurons dying at the lesion site cannot be replaced. In recent years, growing attention has been focused on in vivo glia-to-neuron reprogramming [141,142,143]. It has been established that astrocytes are particularly promising candidates for reprogramming into neurons, as they maintain some of the original patterning information from their radial glial ancestors [142,144]. Using exactly defined transcription factors in vitro [145,146], astrocytes have been successfully reprogrammed into different types of functional mature neurons. Su et al. [141] examined the possibility of reprogramming endogenous non-neural cells, such as scar-forming astrocytes into neurons in the adult mouse spinal cord. They indicated that a high-mobility group of DNA-binding domain transcription factor, SOX2, known to be essential for specification and/or maintenance of progenitor identity [147,148] uniquely converted resident astrocytes into doublecortin (DCX+) neuroblasts and microtubule associated protein 2 (MAP2+) mature neurons. Their data suggest that in the adult spinal cord, a threshold of SOX2 expression is required to induce cell fate change. When mice after Th8 hemisection were injected with hGFAP-GFP-T2A-SOX2 lentivirus, all the induced DCX+ cells also expressed green fluorescent protein (GFP), indicating an origin from virus-transduced cells. Approximately 3–6% of GFP+ cells surrounding the core viral injection sites were reprogrammed by SOX2 to become DCX+ cells between 4–8 wpi. DCX+ cells were also positive for neuronal marker TUBB3 (Tubulin Beta 3 Class III). These data indicate that neurogenesis can be induced by SOX2 in an injured environment of the adult spinal cord. These results also show that SOX2-induced adult neurogenesis can generate mature neurons with features of GABAergic (gamma-Aminobutyric acid) interneurons in injured valproic acid (VPA)-treated spinal cords. Although the number of converted neurons was low, the authors found that new neurons were capable of forming synapses with preexisting ChAT+ motor neurons, suggesting potential integration into the local neural network of the injured spinal cord. Recently published data have shown that spinal cord-derived adult astrocytes express a high level of NOTCH1 signaling which is responsible for neuronal stem cell maintenance and neurogenesis in the embryonic as well as the adult brain [149], and they are not susceptible to neuronal reprogramming [143]. These findings indicate that further in vivo studies are necessary to enhance the reprogramming process and to obtain neurons with appropriate subtype identities and projections which are required for functional recovery after SCI.

Over the past decade, a direct parenchymal injection of viral vectors (lentivirus, retrovirus, adeno-associated virus) has become frequently used for in vivo reprogramming, achieving a broad range of reprogramming efficacy and neuronal survival [142,150,151]. Although effective in delivering these viruses into the spinal parenchyma, the invasive nature of this approach limits the number of injections that can be performed. A novel subpial delivery technique permits widespread transgene expression within the spinal parenchyma in adult pigs, rats and mice [152,153] and does not require direct spinal cord tissue needle penetration. This delivery technique can be used in experimental, pre-clinical and human clinical studies to regulate genes of interest in specific spinal cord segments and/or in the projection of motor and ascending sensory axons. This novel approach is extremely effective in achieving trans-spinal occupation by grafted cells, particularly in the treatment of SCI characterized by multisegmental degeneration [154,155,156].

## 12. The Effect of Weak Long-Term Electrostimulation on Spinal Cord Functional Recovery

One of the major limiting factors for functional regeneration after traumatic SCI is the inability of damaged axons to re-establish their interconnections with target fibers on the opposite side of the lesion. Application of a weak electric field over the injury site is one of the methods enabling the regrowth and proper alignment of damaged nerve fibers. Extracellular electric fields produced by weak electrostimulation presenting the voltage gradient within tissue might provide the necessary stimulus directing astrocyte behavior after CNS injury and gradually dissolving glial scar integrity. It has been shown previously, that electric fields affect directly-induced cellular behaviors, e.g., migration [157,158], proliferation [159,160], differentiation [161,162] and morphology [163,164] among the variety of ectodermally and mesodermally-derived cell types [165,166].

In the past, Borgens et al. [167] demonstrated that glial cells, astrocytes in particular, are able to respond to weak electric fields. These authors showed that rat cortical astrocytes oriented themselves along the applied voltage gradient in experiments in vitro. Moriarty and Borgens [168] also reported that applied voltage reduced the number of astrocytes accumulating at the site of SCI and suppressed the extension of astrocytic processes within the lesion site. On the other hand, the other major components of the inhibitory glial scar, macrophages, do not seem to be affected by exogenous electric gradients [169]. To enhance the regeneration of both ascendent and descendent neural pathways simultaneously, an oscillating electric field stimulation (OFS) technique, which periodically (every 15 min) changes the polarity of the electric field, has been developed. The application of a weak oscillating field current over the lesion site of SCI mimicking the polarity guidance in the developmental stages in CNS, has been shown to promote regeneration of injured axons, stimulating them to grow across the injury site [170].

A miniature electric stimulator (50 µA) with oscillating electric field (OSF) was used in rat SCI experiments in vivo [171,172]. Spinal cord trauma caused considerable increase in activated forms of astrocytes, a typical feature of the ongoing inflammatory reaction four weeks post-SCI. The greatest accumulation of reactive astrocytes was observed in the areas of the dorsal and lateral spinal funiculi. This observation correlated with histopathological findings indicating the greatest tissue and myelin loss in these white matter regions. Stimulated animals (SCI + OFS) showed a significantly lower number of activated astrocytes and larger area of preserved spinal cord tissue compared to their state after SCI, with higher locomotor activity of the hind limbs and earlier onset of spontaneous urination [171].

Zhang et al. [173] and Jing et al. [174] proposed that electrical stimulation might promote spinal tissue integrity and contribute to remyelination after SCI via improved differentiation of oligodendrocyte precursor cells. A similar beneficial outcome was reported in a study of epidural stimulation after SCI, where the electrostimulation upregulated myelin basic protein mRNA levels and reduced oligodendrocyte loss by promoting their differentiation and inhibiting apoptosis [157]. Long-term (eight weeks) epidural stimulation with OFS applied immediately after spinal trauma significantly reduced oligodendrocytes loss and promoted their density in the areas of the greatest tissue damage [172]. Similarly, by reducing reactive astrogliosis and glial scarring, where the oligodendrocytes are greatly affected by inhibitory components of the glial scar [175], they were able to migrate towards the lesion site and initiate remyelination. According to these data, we suppose that OF stimulation applied after SCI could provide a more hospitable microenvironment either for neurons or glial cells by triggering the regenerative processes in the acute phase of injury.

The exact mechanism responsible for axonal and glial regeneration in response to applied electrical stimulus is not yet fully understood. Axonal growth after electrical stimulation has been presumed to be mediated by membrane receptors and secondary messengers such as adenylcyclase and interaction with other physiological neurotrophins presented in the CNS [176,177]. Electrostimulation has also been shown to enhance the expression of regeneration-associated genes—*RAG*s [178,179], which are functionally required for neural recovery [180,181].

## 13. Rehabilitation-Comprehensive and Effective Therapeutic Strategy after SCI

Changes at molecular and cellular levels could provide new insights into mechanisms by which exercise has a positive impact on functional deficits occurring after SCI. Since positive effects of physical activity and exercise have been clearly demonstrated in patients after traumatic SCI [182], rehabilitation and exercise appear to be the most effective non-invasive post-SCI therapeutic strategy. In addition to strengthening muscle mass, rehabilitation is effective in endogenous stimulation of growth factors [183,184,185]. Previous experimental studies have shown that various forms of rehabilitation (treadmill, swimming, physiotherapy) significantly supported functional spinal cord regeneration [186], and that physical training is important for regaining motor and sensory function after SCI.

Exercise is no longer strictly a tool for rehabilitation and there are many exciting aspects of this therapy which remain to be explored, such as the time post-injury when exercise is best initiated, the most appropriate intensity, duration and frequency, and the best use of task-specific and non-task specific training for recovery of multiple functional modalities [185]. Novel data show that treadmill training (six weeks) prior to SCI markedly increased the activity of phospholipase Cγ/ protein kinase C (PLCγ-PKC) signaling at both transcript and protein levels at and around the lesion site. Similar effects were seen in expression of phosphoinositide 3-kinase/ protein kinase B (PI3k/Akt) and Ras/extracellular signal-regulatedkinase 1/2 (Ras/Erk1/2) signaling responsible for cell survival and regeneration [187]. Recently, Zhang et al. [188] demonstrated that inhibition of aldose reductase, which plays a key role in a number of inflammatory diseases, significantly attenuated LPS-induced activation of PLC-PKC pathway, and that such inhibition can work as a switch, which regulates microglia by polarization either to M1 or M2 activity after spinal trauma. Moreover, Mohanraj et al. [189] found that Trehalose-6,6′-dibehenate (TDB) could inhibit LPS-induced inflammatory response through the PLC-γ1/PKC/ERK signaling pathway and promote microglial polarization towards the beneficial M2 phenotype via the phospholipase Cγ/calcium/calmodulin-dependent protein kinase kinase-β/activated protein kinase (PLC-γ1/calcium/CaMKKβ/AMPK) pathway. These results indicate that molecular analysis of the signaling pathways responsible for survival, plasticity and neuroregeneration after assisted long-term post-SCI training could be very useful, and could be used in further experimental post-SCI rehabilitation strategies.

## 14. Conclusions and Future Direction

In recent years, substantial advances have been made in identifying multicellular interactions and their molecular cross-talk that shape the response to SCI. A detailed knowledge of glial-neuronal interactions within the lesion microenvironment could be very effective in revealing promising therapeutic strategies. In acute and subacute SCI stages, the spinal cord possesses an underestimated and underexploited plasticity. Indeed, early modulation of inflammatory response after SCI, regulation of glial-neuronal cross-talk within the epicenter of injury and in its vicinity, and time-dependent transformation of reactive microglia and astrocytes into their neuroprotective phenotypes are crucial steps towards more successful treatment of traumatic SCI. Another very important way to decrease inflammatory cytokines and mitigate robust inflammation within the lesion epicenter is modulation of SCI-induced gut dysbiosis. Future research should focus on investigating the intraspinal and systemic SCI pathology.

Although to date there is insufficient evidence to draw meaningful conclusions regarding the effectiveness of post-SCI electrostimulation and/or rehabilitation in improving physical capacity and/or functional outcome after moderate SCI, no study has reported their negative impact. Taking into account the complexity of secondary damage and a limited ability of endogenous regenerative processes, future strategy for effective therapy consists of appropriate combination of experimentally promising individual treatment approaches, and its translation to the clinical practice.

## Data Availability

Not applicable.
